# Comment on Karthikeyan et al. Concordance between In Vitro and In Vivo Relative Toxic Potencies of Diesel Exhaust Particles from Different Biodiesel Blends. *Toxics* 2024, *12*, 290

**DOI:** 10.3390/toxics13030174

**Published:** 2025-02-28

**Authors:** Katherine R. Landwehr, Alexander N. Larcombe

**Affiliations:** 1 Occupation, Environment and Safety, School of Population Health, Curtin University, Perth, WA 6845, Australia; katherine.landwehr@thekids.org.au; 2 Respiratory Environmental Health, Wal-yan Respiratory Research Centre, The Kids Research Institute Australia, Perth Children’s Hospital, Nedlands, Perth, WA 6009, Australia; 3 School of Human Sciences, University of Western Australia, Crawley, WA 6009, Australia

Biodiesel exhaust toxicology is a difficult field of study, for which there is a paucity of literature, despite decades of research into the subject [1,2,3,4,5]. The majority of biodiesel-focused publications assess fuel creation methods or the exhaust profile and impacts on the engine, not the downstream toxic effects of exhaust exposure [1,2,3,4,5]. This is likely because the in vitro and in vivo biological testing needed to appropriately assess health effects is complex, time consuming, expensive, and spans multiple fields of research, thereby requiring experts from a wide range of disciplines that typically do not collaborate.

We recently read the study by Karthikeyan et al. [6] assessing the toxic impact of three different 80% diesel/20% biodiesel (B20) particles: canola B20, soy B20, and tallow B20. The authors collected exhaust particles on PFTE filters and exposed cells from the J774A.1 mouse macrophage cell line to the generated particle suspension, as well as performing intratracheal instillation of particles in BALB/c mice. The authors assessed a range of downstream effects for both the in vitro and in vivo models, including cytotoxic endpoints (e.g., lactate dehydrogenase (LDH) release, resazurin reduction (CTB), and ATP assay), cytokine responses, bronchoalveolar lavage (BAL) cell counts, lung and heart mRNA changes, and serum markers. The main findings were that particles from ultra-low sulfur diesel (ULSD) and canola B20 were more potent and caused a greater range of toxic effects than soy and tallow B20, a finding that conflicts with multiple studies that the authors have neglected to cite [7,8,9,10,11,12]. Overall, the authors appear to have missed a proportion of the relevant, recent literature in their manuscript, with only six citations being from the last five years, and over three-quarters of the cited sources being published ten years ago or earlier. This is of importance due to changes and improvements in exhaust after-treatment technologies in recent years, which will significantly impact the toxicological outcomes. This issue is compounded by the fact that the authors used an engine compliant with the emission standards of 2004, meaning that (at best) it is Euro III, and therefore permitted to produce far more particulate matter (PM), oxides of nitrogen, and other pollutants compared with a modern engine. The data produced by Karthikeyan et al. are therefore primarily of relevance to engines > 20 years old, or engines used in jurisdictions with less stringent emissions standards.

That said, the authors should be commended for expanding the knowledge on the health effects of biodiesel exhausts, particularly for their comparison of different types of biodiesels within the same study, when many previous articles assess only one type and make general assumptions about biodiesel as a whole based on their findings [13]. However, the most important purpose of in vitro and in vivo health effect analysis of biodiesel exhaust is to extrapolate the findings from models to predict the potential health effects of human exposure. To that end, a “real-world” relevant exposure methodology should be employed where possible. Isolating the particles from diesel exhaust is less real-world relevant than using whole exhaust exposure, and can substantially impact outcomes [14,15,16]. We acknowledge that whole exhaust exposure can be difficult and expensive to achieve without access to the appropriate equipment and facilities. Karthikeyan et al. [6] collected exhaust particles on PFTE filters to generate particle suspensions for downstream analyses. This approach, while failing to account for the health effects of the exhaust gases, does provide more control over the exact dose of PM used for each exposure. However, the authors failed to specify the pore size used for the filters. Both diesel and biodiesel exhausts contain a spectrum of particle sizes, ranging from ultra-fine (<100 nm in size) to coarse (<10 µm in size). In diesel exhaust, ~90% of particles by number are under 100 nm in size [17,18], and in biodiesel exhausts, the median particle size is generally smaller [7,19]. If the PFTE filters used did not have the correct pore size, the majority of particles may not have been collected, which skews the results of the resulting exposure’s health effects [14]. Additionally, diesel exhaust PM agglomerates on filters to produce an artificial particle size spectrum [15], which is unlikely to be reversed with sonication and “rigorous pipetting”, as used by the authors. It must be acknowledged that this is an important limitation in assessing exhaust toxicity both in vitro and in vivo. If Karthikeyan et al. have access to the particle spectra from their study that compare the whole exhaust to the particles in suspension, this information should be provided to allow for accurate comparisons between the different exhaust types. This is to verify that observed differences in toxicity are not caused by changes in particle size, and thus the number of particles collected on the filters.

A second limitation of the study by Karthikeyan et al. is their choice of in vitro model. Using immortalized human cell lines will always come second to primary human samples [20,21], and Karthikeyan et al. [6] chose to use a mouse macrophage cell line to assess toxic effects instead of a human one. Human macrophage cell lines are available [22], which brings the relevance of using a mouse macrophage cell line into question, especially considering the differences between human and mouse macrophages [23,24]. The isolation of peripheral blood monocytes directly from human blood samples is now a routinely performed procedure, and inexpensive kits are available [25]. These isolated cells can then be further derived into macrophages [25]. Karthikeyan et al. [6] do not appropriately address this limitation in their article. Additionally, although it is not overtly stated, Karthikeyan et al. [6] appear to have used a submerged cell culture model. As per our previous comments, this type of model is considerably less relevant than employing a fully differentiated culture and exposure at the air–liquid-interface (ALI). Such exposures are commonly conducted and have been shown to produce more realistic exposure toxicology data than submerged cultures [26,27]. In the study by Karthikeyan et al. [6], the use of an ALI culture model is likely moot, due to the initial choice of exposing a mouse macrophage cell line to collected particles in suspension.

Finally, Karthikeyan et al. [6] present a very limited array of in vivo outcomes. There are no lung functional outcomes, and while the authors state that “differential cell counts” were performed, only neutrophil numbers are presented. Exposure to both diesel and biodiesel exhausts elicits changes in other cell types in vivo [8], raising questions as to why these data are not fully presented. Related to this, it is likely that using a lavage volume of 5 mL per mouse (which is >5 times more than usual [28]) would produce spurious results through the mechanical over-inflation of the lungs. We assume that this is an error, and that the authors meant 0.5 mL.

In summary, while the work of Karthikeyan et al. [6] should be commended for broadening the narrow field of research into the effects on human health of biodiesel exhausts and directly comparing several types of blended biodiesel exhausts, there are several limitations of the study design that need to be considered when interpreting the data. Until those questions are addressed, caution should be used in interpreting the results, and ultimately, the conclusions drawn.

## Data Availability

No new data were created or analyzed in this study. Data sharing is not applicable to this article.
